# Acquiring Respiration Rate from Photoplethysmographic Signal by Recursive Bayesian Tracking of Intrinsic Modes in Time-Frequency Spectra

**DOI:** 10.3390/s18061693

**Published:** 2018-05-24

**Authors:** Mikko Pirhonen, Mikko Peltokangas, Antti Vehkaoja

**Affiliations:** BioMediTech Institute and Faculty of Biomedical Sciences and Engineering, Tampere University of Technology, 33720 Tampere, Finland; mikko.peltokangas@tut.fi (M.P.); antti.vehkaoja@tut.fi (A.V.)

**Keywords:** photoplethysmography, respiration, time-frequency analysis, particle filters, synchrosqueezing

## Abstract

Respiration rate (RR) provides useful information for assessing the status of a patient. We propose RR estimation based on photoplethysmography (PPG) because the blood perfusion dynamics are known to carry information on breathing, as respiration-induced modulations in the PPG signal. We studied the use of amplitude variability of transmittance mode finger PPG signal in RR estimation by comparing four time-frequency (TF) representation methods of the signal cascaded with a particle filter. The TF methods compared were short-time Fourier transform (STFT) and three types of synchrosqueezing methods. The public VORTAL database was used in this study. The results indicate that the advanced frequency reallocation methods based on synchrosqueezing approach may present improvement over linear methods, such as STFT. The best results were achieved using wavelet synchrosqueezing transform, having a mean absolute error and median error of 2.33 and 1.15 breaths per minute, respectively. Synchrosqueezing methods were generally more accurate than STFT on most of the subjects when particle filtering was applied. While TF analysis combined with particle filtering is a promising alternative for real-time estimation of RR, artefacts and non-respiration-related frequency components remain problematic and impose requirements for further studies in the areas of signal processing algorithms an PPG instrumentation.

## 1. Introduction

Respiration rate (RR) acquisition constitutes a vital part of clinically fundamental physiological measurements, as RR is one of the first parameters responding to decline in patient status. Accordingly, a large variety of instrumentation designs and mathematical algorithms have emerged as a response to occupy this wide field of physiological sensing. Due to obtrusive nature of many currently used measurement methods, RR tracing imposes discomfort for patients, and thus requires consideration of the necessity of its monitoring. To make RR monitoring more available as a general physiological parameter, new sensing modalities and algorithms are widely studied. Photoplethysmography (PPG) is a minimally obtrusive, optical technique used for monitoring changes in blood flow dynamics, traditionally adopted in the measurements of blood oxygen saturation and heart rate. Pioneering works by Lindberg et al. [1] and numerous others thereafter have shown the potential of PPG in assessing respiration events, which have been shown to modulate the signal waveform in both, amplitude and frequency. Prospects for the integration of RR estimation in inexpensive, existing instrumentation, such as pulse oximeter, have been followed by efforts in novel algorithm design on assessing both frequency and time domain features of the signal waveform. Several frequency components are often present within the range of assumed, plausible respiration frequencies, causing challenges to choose the one resulting from respiration. In addition, extracting the RR from the PPG signal is everything but straightforward since PPG is highly sensitive to motion and ambient artefacts [2].

We have approached the algorithm development side in the RR estimation from PPG. In general, low-frequency respiration modulations in PPG are short-term, being easily masked by interference. Their strength is also subject to variation both by measurement location and PPG hardware used. Therefore, we have studied constricting the weak PPG signals in time-frequency (TF) plane to allow more accurate representation of modulation properties than with conventional methods. In addition, we propose following the weak, but sharply represented signal by adding a recursive Bayesian tracking, a particle filter, in the signal analysis. In this paper, we compare different TF calculation methods, and evaluate the effect of particle filtering in the accuracy of the RR estimation results.

### Related Work

As a variety of methods have been developed for respiration rate estimation from a PPG signal, our recapitulation of prior work focuses on those works with methods similar to that which we used. Comprehensive reviews and potential algorithm designs in PPG-derived RR estimation have been compiled in [3,4]. Synchrosqueezing is a recently introduced tool for identifying oscillatory modes from multicomponent signals. This method for TF reassignments has been presented in [5,6,7] and expresses a similar approach to a method known as empirical mode decomposition (EMD), discussed in [8]. In this study, we have compared short-time Fourier transform and three TF synchrosqueezing techniques that all emerge either from wavelet-based or Fourier analysis. Previously, wavelet-reallocated synchrosqueezing transform has been suggested for PPG-derived RR assessment in conjunction with cost function derived ridge extraction in [9]. Synchrosqueezing has also been applied for the estimation of instantaneous heart rate from PPG signal [10]. Particle filtering in PPG-derived RR extraction has been proposed previously in combination with autoregressive (AR) signal modelling in [11,12]. Besides synchrosqueezing, also other novel and efficient TF analysis methods, such as variable frequency complex demodulation have been proposed for RR estimation [13]. In addition, similar approaches have been proposed in ECG framework [14,15], but combinations of TF reassignments and particle filtering have been disregarded. We are not aware of prior works assessing particle filtering for RR estimation in PPG analysis in combination with synchrosqueezing.

Synchrosqueezing essentially allows shorter time scale detection of the respiration frequencies than the conventional methods, which require longer time windows to obtain similar frequency resolution. A study on two public databases has shown that instantaneous RR, essentially respiration frequencies expressed at very short time scale, can be accurately extracted by synchrosqueezing [10]. In that study, an algorithm called ‘deppG’ was used to show that synchrosqueezing provides a major improvement to extraction of respiration components, vastly outperforming many contemporary methods. DeppG involves a de-shaped synchrosqueezing which is described in more detail in [10]. Our proposed algorithm is similar in the sense that it constructs the respiration components by a synchrosqueezing method, but we also tested if RR tracking by particle filtering improves the results.

## 2. Materials and Methods

Conventionally, most of the PPG based RR algorithms are comprised of three operational phases [3,4]

(1) preprocessing of the signal,
(2) extraction of the respiratory component, and
(3) determination of the respiration rate.

These phases may be followed by an additional option for the post-processing for example by filtering the result or by fusing the results produced by several methods. Decision on the approach essentially includes the choice of the modulated quantity, which is commonly amplitude, frequency, or baseline of a signal. Various algorithms may be formulated as combinations of different techniques in each of the phases, in which the RR information may arise from frequency or feature (morphological) domain detection. Fusion options include, but are not limited to, combining the information from different modulation sources.

In this paper, we follow the aforementioned phases in the algorithm construction for estimating the RR from TF spectral representations. The preprocessing step of the signal consists of the extraction of amplitude variability information, also known as respiratory-induced amplitude variation (RIAV). The preprocessing is followed by the selected frequency mode decomposition methods based on synchrosqueezing or Fourier transform, which represents phase 2. By the general assumption, the largest spectral peak within the appropriately chosen region of interest (ROI) represents RR, commonly assigned below 1 Hz, corresponding to 60 breaths per minute (bpm). After decomposition, particle filtering, a recursive Bayesian tracking algorithm, is applied to estimate the path of the sought, intrinsic respiration mode and to extract the instantaneous respiration frequency. Tracking the respiration component represents the third phase of the algorithm.

Our RR estimation algorithm begins with the PPG signal variability extraction. This is followed by the spectral estimation of the variability signal content. We have compared four spectral estimation methods:

(1) Short-time Fourier Transform (STFT),
(2) Wavelet synchrosqueezing transform (WSST),
(3) Fourier synchrosqueezing transform (FSST), and
(4) Vertical second-order synchrosqueezing transforms (VSST).

**Figure 1:**
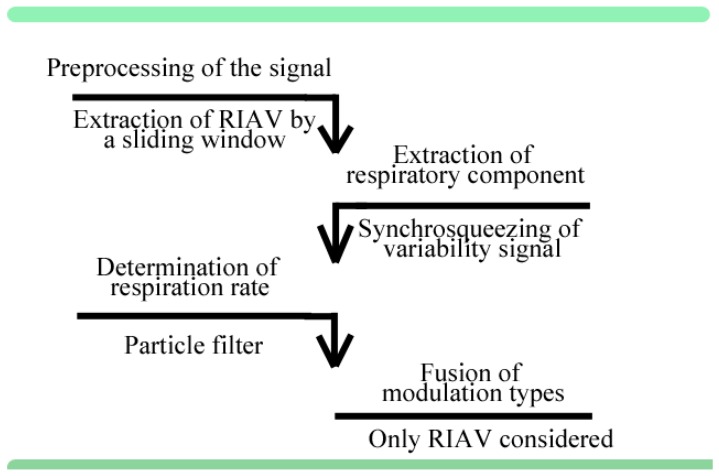
Operational phases of the generic respiration rate (RR) estimation algorithm (the text above the arrows). The methods used in the present study are shown below each arrow. The last step, fusion of different modulation types, is optional and was not implemented in this work since only respiratory-induced amplitude variation (RIAV) was studied.

After spectral estimation, a particle filter is applied for determining the RR. Figure 1 illustrates the flow of the analysis protocol. The analysis steps are described in detail in the following sections. Subsequent particle filtering, a probabilistic Bayesian algorithm, weights the importance, that is, likelihood, of the mode function that is formed by the instantaneous frequency components at their respective points in time providing the estimation for the RR. Both algorithm development and evaluation were implemented in MATLAB^®^ software.

### 2.1. Signal Variability Extraction

In the acquisition process of RR estimates, the initial steps of PPG signal processing have generally followed a similar protocol. The original signals are either low-pass or band-pass filtered with appropriately chosen cut-off frequencies. Next, assessing peaks and troughs of the filtered signal, a threshold mechanism is used to extract signal values according to modulation type studied. The result is a signal that contains variability values as these emerge from selected modulation type. Amplitude, baseline and frequency modulations of the signals contain usable information for the RR estimation as respiration likely affects both amplitude and phase of the PPG-signal.

In the present study, we have implemented pulse amplitude variation of the PPG signal as the feature for RR estimation. While it is true that the amplitude is affected by various sources of noise and blood oxygen saturation level (SpO_2_), we note that these changes likely occur at lower frequencies and therefore will not significantly affect the results. Movement artefacts would definitely have a large effect but the data set used has been recorded in static condition.

The initial step of our method is to filter the signal with a 6th-order Butterworth band-pass filter having lower and upper cut-off frequencies at 0.06 Hz and 25 Hz using forward-backward filtering. This frequency band preserves the primary entities of interest in the waveform. Maximum and minimum of each individual pulse waves were extracted by applying adaptive threshold in a sliding window of uniform length (3 s) to the first derivate of the PPG. Zero crossings of the first derivate were utilized in the detection of peaks and troughs from the original signal. The output of this preprocessing step is a time series of the amplitude values of the PPG signal labeled as the variability signal PPG_RIAV_, where variability is characterized by changes in amplitude of consecutive sets of peaks and troughs in the original PPG waveform. 

As some considerable artefacts may occur in PPG signal and thus also in PPG_RIAV_, we removed maxima or minima outliers representing markedly deviating values, differing more than 1.3 times the 99th percentile of PPG_RIAV_ values, and replaced those by appropriate spline-interpolated values (not-a-knot end method). Finally, the PPG_RIAV_ was interpolated to the sampling frequency of 1.5 Hz. For a frequency component analysis, the ROI was set in range of 0.1–0.75 Hz in accordance with Nyquist sampling theorem, corresponding to 6 bpm–45 bpm as has been proposed earlier in [12,16]. This frequency band corresponds to the range of both healthy and disordered RRs in adult subjects.

### 2.2. Expressing Signal in Its TF Spectrum

Having selected appropriate limits for ROI in the RR estimation, one may continue to observe well-separation conditions (i.e., the combined time and frequency distinction) [17] of the intrinsic frequency modes within the ROI limits. Notably, respiration is not the only intrinsic frequency component within the predetermined region. At low frequencies (0.04–0.15 Hz), two notable contributors affect the PPG signal: Mayer waves [18] and regulatory responses by the sympathetic tone of the autonomous nervous system [13]. Distinction of the RR at these frequencies may be thus challenging. Another challenge is the suppression of measurement artefacts, and especially the artefacts caused by movements or changes in ambient illumination conditions. These interfering components appear as individual frequency carriers. According to the well-separation conditions of the TF representations, these modes may share a superposition at the same frequency or, in case of wavelet-based methods, the same mother wavelet, causing spectral smearing (or, ‘coloring’). The ability of a certain TF representation to extract the RR component may depend on the separation conditions of the artefacts and respiration modes, and these properties therefore greatly affect the robustness characteristics of each proposed TF reassignment method. For methods applying synchrosqueeze mapping, this reflects on their ability to suppress uncertainty principle of frequency and time domains [19], while for linear methods this is limited, as discussed in Section 2.3. In the evaluated approaches, the respective frequency instant of the signal is determined according to reassignment formulae of the TF transforms given in the following sections. Justification for synchrosqueezing is that the reassignment mapping of the frequency and the group delays of the signal waveforms provide us with clear values of the respiration carrying instantaneous frequencies.

### 2.3. Short-Time Fourier Transform

In order to perform adequately in varying respiration frequencies, tracking algorithms need precise information on frequency and time components of the signal. Short-time Fourier Transform is the basic method often applied for creating a spectrogram, which shows the frequency content of PPG_RIAV_ as a function of time. STFT is a widely used method in signal processing in the last decades due to the ease of application and collective understanding of theory behind its formulation. In all simplicity, STFT is a collection of fast Fourier transforms (FFTs) evaluated as successive blocks of the time domain signal weighted by a window function. The STFT results in a complex-valued matrix, which can be visualized as a spectrogram. This allows for compact representation of stationary-assumed components as quasi-non-stationary up to the temporal resolution in which STFT is applied. The spectrogram of the STFT in continuous-time is mathematically given as

$${\left|V_{f}^{g}\left(\eta ,t\right)\right|}^{2}={\left|f,g_{\eta ,t}\right|}^{2}={\left|\int_{\mathbb{R}}f\left(\tau \right)g\left(\tau -t\right)e^{-2i\pi \eta \left(\tau -t\right)}d\tau \right|}^{2}$$

where *f* and *g* are the signal and the window function, respectively, both defined in L^2^(ℝ) [20].

Despite the aforementioned, this method has a major drawback imposed by the Heisenberg-Gabor uncertainty limit [19]. For the STFT, this is observed as a difficulty in achieving sufficient resolution in both frequency and time domain, a difficulty emerging from uncertainty theorem and expressible by limit *∆f∆t* ≥ (4*π*)^−1^ [19]. In other words, it is not possible to express unambiguously in TF spectra the instance at which specific, high-resolution frequency component deviates in temporal plane. One is thus obligated to balance between frequency and temporal resolution of the estimated TF spectrum. While RR estimation using TF representations assumedly benefits from sufficient time resolution and fine details of frequency, it is questionable, whether the performance of the STFT is adequate for obtaining satisfactory results in cases where fast response to the changes in RR is desired. However, the method is likely to preserve a respiration trend to certain degree and can therefore be usable if fast response is not required.

STFT was constructed in this work to have a time resolution of 90 windows per minute, with Gaussian window function as proposed in [20], and with the window parameter α = 1/8. PPG_RIAV_ signals were expressed as STFT spectrograms and the localized time and frequency components were used in later determination of RR by a tracking method.

Due to uncertainty limitations, STFT exhibits smearing of TF components. This means that localization of main frequency components is challenging, and multiple frequency modes may overlap in the TF spectra. Synchrosqueezing addresses this problem by remapping, or ‘squeezing’ frequency information so that frequencies can be readily distinguished as separate entities without compromises in time resolution. There are numerous synchrosqueezing transforms that utilize different formalities to reassign sharper presentations for TF spectra. We have selected three of those, namely: wavelet synchrosqueezing, Fourier synchrosqueezing, and vertical second-order synchrosqueezing transform and applied them to PPG_RIAV_ signals to observe how they improve the tracking of RR.

### 2.4. Wavelet Synchrosqueezing Transform

The major drawbacks in the TF resolution of the STFT may be addressed by the introduction of wavelets. The wavelets are a wide library of mathematical constructs that approximate the signal by relieving the frequency or time resolution from uncertainty principles by applying a scale factor. However, the linearity of wavelet transforms introduces smearing, which is an inherent artefact in the mathematical basis of separation conditions concerning frequency components. Thus, the wavelet transform results in inadequate choice of supports within two closely spaced intrinsic mode functions. Wavelet synchrosqueezing transform [5] is a nonlinear transformation and extracts specific intrinsic modes of the signal by reassigning the signal values (energy) in frequency domain by minor time shifts to demonstrate the presence of oscillatory components within the signal frame [20]. In addition, synchrosqueezing transform is invertible and the analysis of the signal modes allows for relatively simple extraction and reconstruction of the signal properties. In the RR analysis, the distinction of individual oscillatory modes by synchrosqueezing increases the accuracy in the estimation of instantaneous frequency ridges compared to linear transforms.

Continuous wavelet transform (CWT) using analytic Morlet wavelet with 48 voices per octave was applied to PPG_RIAV_ signal, and then, preserving the time resolution, instantaneous frequency information was extracted by frequency reassignment in TF plane. Detailed mathematical theory behind WSST is described in [5].

### 2.5. Fourier Synchrosqueezing Transform

While WSST uses CWT to sharpen the frequency components in the signal, Fourier-based methods ‘squeeze’ the frequency components of the STFT spectra. Fourier synchrosqueezing transform [20,21,22] is a distinct Fourier-based TF analysis method analogous to the wavelet synchrosqueezing. Adapting well to linear or sub-linear modulations, FSST provides synchrosqueezing derived STFT methodology, therefore being also known as ‘STFT-based synchrosqueezed transform’. FSST provides a convenient method for multicomponent signal decomposition. It remains considerably accurate in low frequencies, being constrained by second-order phase expansion negligence, that is, weakness in non-trivial modulations in higher frequencies [21]. Differing from WSST, which projects its frequency resolution from a wavelet scale value, FSST follows linear reconstruction, allowing constant phase separation for the representation in time domain. Furthermore, the frequency resolution in FSST is independent of frequency. Another difference between the two methods follows from the assumptions of the modulation origin, namely on the source of instantaneous frequency in their estimates. Accordingly, FSST reconstructs the instantaneous frequency ridges as they were linear, whereas WSST follows a different, wavelet estimate [21] that is logarithmic in its frequency resolution.

Like WSST, the frequency components of PPG_RIAV_ signals in FSST were reassigned and expressed in TF spectra. STFT was applied to the signal studied and the synchrosqueezed transform was obtained using phase transform as described in [21]. Fourier transform applied in this work used remapping, as proposed in [20], to the STFT described earlier.

### 2.6. Vertical Second-Order Synchrosqueezing Transform

In addition to previously mentioned transforms, we also studied a more rigorous method that corrects some of the problems that FSST has with non-linearities and studied if the tracking can be improved with the corrected TF spectra. One of the key requirements for a synchrosqueezing transform to operate under presence of multiple intrinsic frequency components is the fulfilling of the superposition-separation condition, namely the separation between frequency ridges and the mother wavelet or window. Additionally, instantaneous frequencies may exhibit harmonics, which under certain conditions may overlap in the frequency domain. During RR estimation, the separation conditions may be only weakly fulfilled due to varying power of the modulation components acting on the PPG signal. Under certain conditions, mainly frequency-modulated signals may be tracked inadequately with FSST. Vertical second-order synchrosqueezing transforms (VSST) [20,23] have been proposed as an alternative to FSST to overcome its failure to establish modulation with noisy environments and they are closely linked to STFT. The VSST estimates the second-order expansion of the phase under conventional Fourier analysis. While FSST (effectively a first-order STFT synchrosqueeze) neglects the second-order phase, VSST provides more complete and less constrained transform, which gives capacity to respond to non-linearities. For this reason, rigorously modulated signals, and thus instantaneous frequency estimates, are expected to be characterized by less noise and to be observed more distinctly. Nevertheless, the method still imposes restrictions on amplitude modulations of respective modes, which in RR estimation may prove less applicable for our purposes, especially in the case of emerging amplitude modulation. VSST also requires us to make an a priori determination of Gaussian window properties (as this method is based on FSST and STFT). This may prove to be a challenge of trial and error with possible variations emerging among the subjects.

Mathematical basis and the nature of application proposed for this method has been given in [20,23] and was performed with parameters identical to STFT and FSST. Like for the other reassignment methods, PPG_RIAV_ signals were represented as TF spectra to provide the tracking algorithm with enhanced frequency resolution. We have implemented these methods as they were presented in [20] to form the spectra.

**Figure 2:**
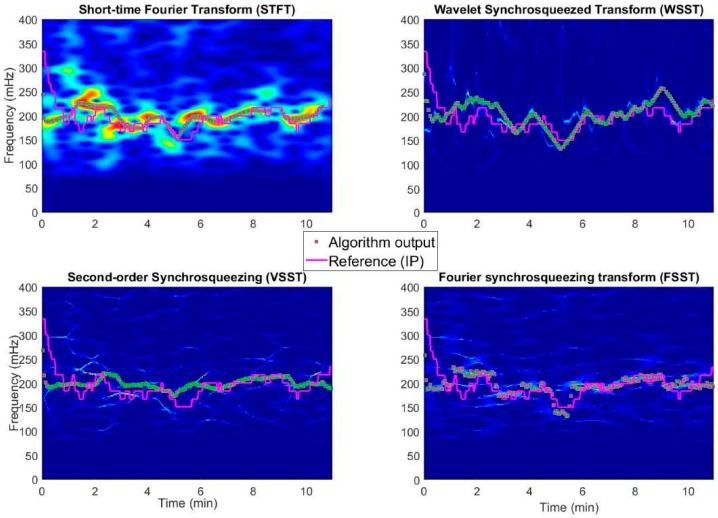
Time-frequency spectra of a subject data produced the evaluated time-frequency conversion methods produce different kind of representations of the analyzed signal. Particle filter response varies according to the spectra-derived state vector input.

Figure 2 shows an example TF representation analysis of a subject data and presents the nature of each TF spectrogram estimation method we have studied in addition to the corresponding particle filter response. This example illustrates how different methods produce different spectrogram responses for the same data. STFT provides a smeared and rather inaccurate representation in frequency and time, yet the method preserves the overall trend. WSST extracts the dominant mode as highly oscillating component, which enables a sufficient response for particle filter for either feature or trend based reconstruction. Both VSST and FSST reassign the modes in a cobweb-like structure of different mode function estimates. In all cases, particle filtering provides reasonably good estimates for the RR.

### 2.7. Particle Filtering

Particle filtering constitutes a set of mathematical formalities, which approximate probabilistic state estimates of a tracking problem in hidden Markov model sense. One of the major advantages in particle filtering is its effective representation of non-linear/non-Gaussian processes while its recursive filtering allows on-line determination of the measured variable. The method relies on finite number of constituents, particles, representing the approximate Bayesian probability distribution of the state of the target system. Estimated posterior density is updated to new state vector positions, which are modelled by random noise contributors with, at least partially known, underlying state of true model extracted from the prior state. Particle filtering is numerical, computationally demanding method to track dynamic systems. Comprehensive reviews of particle filters and their mathematical basis are given in [24,25].

Particle filtering feeds off from state vectors X_s_ by evolution according to **X**_s_ = Ϝ{X_s-1_, n_s-1_}, where X is the particle localization matrix at (discrete) time instances s ∈ ℕ. Prior information at point s-1 is essentially available from the earlier solved characteristics of the system. The Bayesian approach in dynamic system constructs an estimate according to the iterative nature of acquired prior information and noise estimate, modelling a posterior probability density function (PDF), which encompasses the target information. A mathematical operator, such as likelihood, transforms the prior state vector expression (a PDF) to represent an updated solution, which characterizes both updated data and the presence of random noise. In TF spectral analysis, we thrive to understand the evolution of the frequency modulation of the variability signal caused by respiration under discrete time shifts. It is clear that PPG signal modulations in RR range vary greatly during the measurements not only because of respiration but also due to interfering components and are accompanied by varying numbers of modes in TF spectra. Thus, taking the highest spectral peaks of the spectra at each time instant results in grave transients. We create an alternative method by means of particle filtering to extract continuous ridges in the TF spectrogram, and hence approximate instantaneous frequencies, even under the presence of weakly separated intrinsic modes.

The general framework of the particle filters allows for flexibility in the extraction of the RR and relieves the pitfall of following coarse transients habitually present in estimates when using other, simplistic methods that do not consider prior state information. Further, particle filtering provides on-line information of the measured variable and does not require prolonged sequences for ridge estimation. Compared to methods that update the RR estimate in long sliding windows, we update the solution 90 times per minute to illustrate the advantage of using TF spectra and synchrosqueezing.

Particle filtering is robust both in performance and in formulation. In this technique, the number of particles, weighting function, and updating scheme are often adaptable to suit for different applications. As an initial configuration of the implemented particle filter, 100 evenly weighted particles are evenly distributed in the ROI frequencies. In addition, the state vector of the system, including the absolute value of TF spectrogram matrix at time *s*_0_ and the respective frequency components, are extracted and the weights of the particles are updated with this spectral information. The particles thus resemble the PDF of the respiration frequency. Posterior state vectors are then assessed by considering the 5 highest spectral peaks of each time instant, additionally noting that the location of these components frequently includes several modes in the spectra, that is, they are located on different ridges. We formulate the likelihood function as weighted nearest neighbor estimator as proposed in [11], and given as

$$w^{i}\left(n\right)=\exp\left(-\frac{{\left(R^{i}\left(n\right)-p_{nn\left(i\right)}^{a}\right)}^{2}}{2\sigma_{gau}^{2}}\right)\frac{\exp\left(-\frac{{\left(p_{nn\left(i\right)}^{m}-p_{max}^{a}\right)}^{2}}{2\sigma_{w}^{2}}\right)}{\sum_{m=1}^{K_{roi}}\exp\left(-\frac{{\left(p_{nn\left(i\right)}^{m}-p_{max}^{a}\right)}^{2}}{2\sigma_{w}^{2}}\right)}$$

where *R^i^*(*n*) represents particle locations, *p^a^*_max_ is the component angle with highest magnitude and *p^a^_nn_*(*i*) is the nearest frequency angle to particles. Our choice for parameters are *σ*_gen_ = 10^−3^, *σ*_gau_ = 1.5 × 10^−2^, and *σ*_w_ = 7.5 × 10^−3^, and *K*_roi_ = 5. The particle weights thus increase near large spectrum moduli in nearest neighbor sense, diminishing otherwise. Formed likelihood function is presented by a distribution of particles where the mean value of the density function represents the result, that is, the RR.

**Figure 3:**
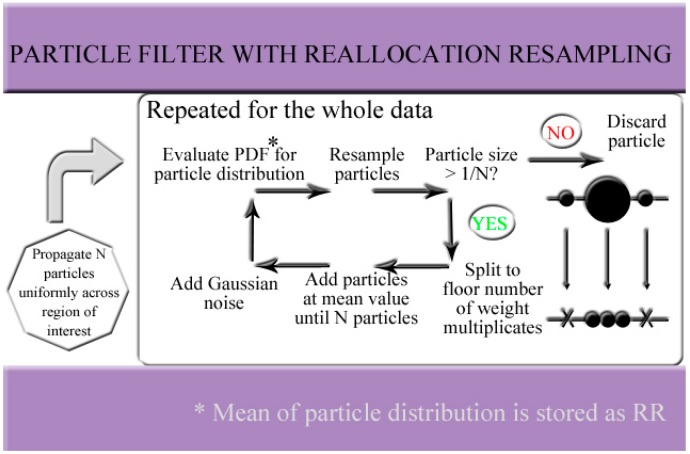
Structure of the proposed particle filter having reallocation resampling.

At each state update cycle, some particles with negligible weights will be discarded as irrelevant. Without being taken care of, the particles would, after a long enough time, converge to a single point in the state that is closest to the largest component of the spectra and later points in time no longer affect the outputs of the filter. This undesirable behavior, generally known as particle degeneracy, is the result of convergence negligence of the weight updates. This diminishes the undesired state regions where spectra components are valued low in weighted nearest neighbor sense and thus considered irrelevant. However, to consider the future responses in RR, the entire ROI should be accessible in practical, dynamically changing inputs. This issue has led to numerous resampling schemes, that is, manners in which new particles are propagated to uphold the number of particles in each updated round of particle filtering. In order to provide a filter construction with small particle distribution variances, we have relied on reallocation resampling in the degeneracy problem, which has an advantage of decreased resampling variance (the new particles are located close to the previous PDF center) compared to traditional resampling [26]. The general structure of the particle filter is illustrated in Figure 3. The particles with greatest weights are preserved according to splitting criteria: in reallocation resampling, a particle is split to floor number of its weight divided by a threshold weight 1/*N*, where *N* is the number of particles, while particles with weights less than the threshold weight are discarded. This results in varying particle set sizes.

**Figure 4:**
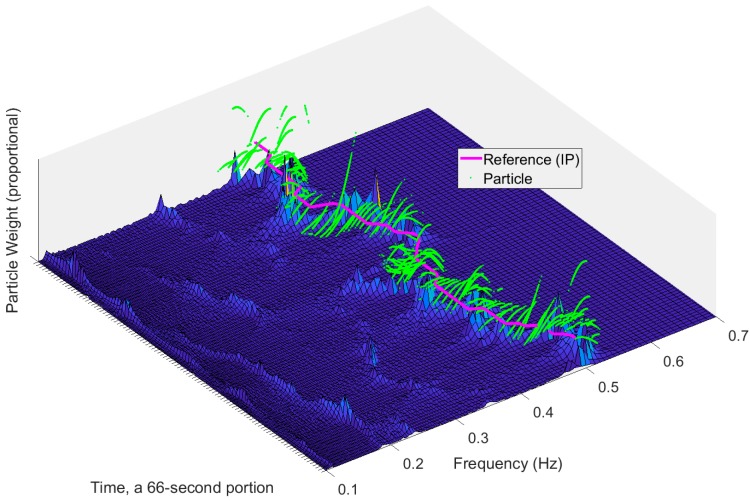
Response of the particle filter to a 66-s segment of subject data in wavelet synchrosqueezing transform (WSST). Particle filter exhibits fast response to underlying signal variations given that time resolution of the system model is adequate.

In absence of signal noise, the particle filter should approximate the correct state, that is, the frequency within the first few iteration rounds. However, PPG and the variability signals derived from it have a complex waveform, sensitive to various artefacts. Accordingly, we may expect the noise and disturbances to mask the correct frequency components in the signal. We know that the correct RR is dynamic and thus subject to changes over time. After the particle filter has converged during the first iterations, small jitter of the particles at each step is essential to allow the filter to follow the changes in the RR. In the implemented particle filter, Gaussian noise was used to propagate the particles at each step. Particle weights are updated so that the sum of the weights is normalized to unity and the resampling is subsequently performed on each individual particle, that is, new particles are propagated in the frequency range as a function of particle location and weights. By obtaining particle filter -derived estimate on every available time instance, it is possible to acquire fast response to changes in the underlying signal dynamics as shown in Figure 4.

### 2.8. Evaluation of the Proposed Method

We have used the publicly available VORTAL dataset [3,4], which includes 39 roughly 10-min infrared PPG measurements on young, healthy subjects at rest (supine) and reference RR acquired both by impedance pneumography (IP) and nasal pressure sensor. In the current analysis, the nasal pressure sensor data was primarily used because this signal appeared to characterize respiration with less erroneous transients than observed in IP signal.

Acquisition of the RR estimates was conducted using the three previously described synchrosqueezing transforms and the STFT. The TF spectra of PPG pulse amplitude signals were computed for each method and the instantaneous RRs were extracted from the particle filter outputs. The extracted results from 39 healthy subjects at rest were compared with the reference (nasal pressure sensor data) provided in the dataset. For equal comparison between the subjects, equal-length signals from each subject were analyzed. The duration of the shortest measurement was approximately 8 min, so the first 8 min of each measurement were selected for the RR estimation. Both reference and PPG-based RR estimates were obtained at a rate of 90 samples per minute. Each frequency output from the particle filter was compared to the respective spline-interpolated reference value.

A poor signal-to-noise ratio of the reference signal caused errors in the reference RR estimate. For this reason, an 8-sample-length median filter was applied to the reference signals. In case the quality of the nasal pressure data was deemed insufficient, that is, it included excessive number of erroneous spikes, corresponding IP reference was used instead. This was the case with three subjects (subjects 21, 22, and 28, in VORTAL data). This issue should be noted in future use of the dataset.

### 2.9. Statistical Methods

Performance of the proposed algorithm was assessed by conventional, statistical metrics: mean absolute error (MAE), median (absolute) error, mean error, and root mean square (RMS) error, which were calculated against the reference RR data. Recall percent is shown to compare the robustness of the methods. In addition, coverage probability CP_2_ percent gives the percent of results falling within 2 bpm error margin. Also, standard deviations of the aforementioned statistical metrics are reported.

To illustrate the accuracies in TF presentations we have also calculated MAE if the highest spectral peak had been chosen instead of applying particle filtering. Further, to illustrate in a way the maximum achievable accuracy with each TF estimation method, we show the result obtained if picking the closest component to RR reference out of the 5 largest spectral components. This is clearly a hypothetical way to compare the accuracy of the methods and is shown only to illustrate that the dataset appeared consistent with relations of PPG signal and RR references.

In addition to the aforementioned metrics, we have visualized the results obtained with the best performing TF method with a Bland-Altman plot. Limits of agreement (LOA) have been calculated as is customary for Bland-Altman plots. We will also show how MAE was divided between different subjects in our study.

## 3. Results

**Figure 5:**
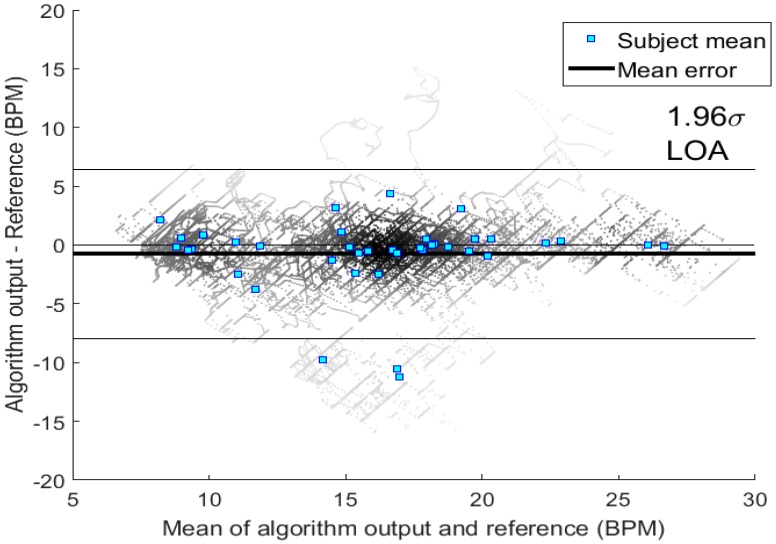
Bland-Altman plot for the WSST. The individual RR-results are represented by their density (Voronoi tessellation). The average values of the individual subjects are shown with blue squares. The three subjects outside the 1.96*σ* limits are subjects 22, 31, and 34.

Table 1 shows the results with standard deviations obtained with different TF representations. According to the results obtained using particle filtering, WSST provided mostly consistent results with mean absolute and median error values of 2.33 bpm and 1.15 bpm, respectively. Figure 5 illustrates the Bland-Altman plot for the results of WSST method with 95-% LOA of [6.5, −8.0] bpm with respective α = 0.05 confidence intervals of LOA as ([6.52, 6.39], [−7.91, −8.04]) bpm. These LOA bounds are similar in magnitude to those for other algorithms as shown in [3], where 270 different combinations of PPG based RR algorithms were assessed. While the most efficient PPG algorithms used time-domain breath detection (as phase 3), we have now observed similar performance in frequency-based methods. Moreover, some of the signal waveforms deemed low in quality were excluded from analysis in [3], while to our advantage we have not labeled waveforms by their quality.

**Figure 6:**
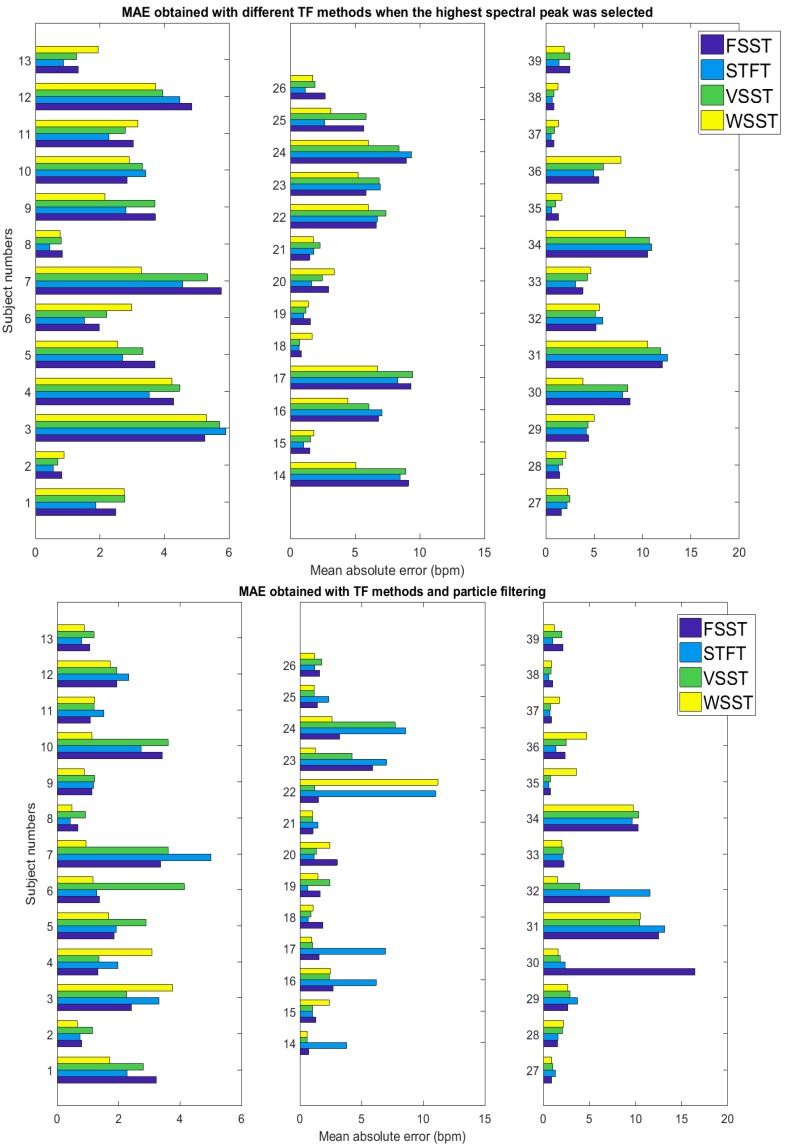
Comparison of mean absolute error between the methods in each subject. Upper: mean absolute error (MAE) for each TF method if only frequency component with highest spectral peak component is selected, disregarding particle filtering. Lower: MAE when particle filtering has been applied.

MAE is illustrated for each subject in Figure 6. From this figure, we observe the differences in MAE values for different subjects with and without particle filtering. The results for subjects 31 and 34 (in VORTAL dataset [3,4]) especially were relatively poor in accuracy, both before and after particle filtering. This contributed to most of the increase in the observed error of the tested methods. Some subjects, such as 22 and 30, produced large errors in one method but not in others, as can be seen in Figure 6. The errors in these subjects, as well as seldom, erroneous, or missing instantaneous frequencies in several other subjects, were commonly characterized by temporary mixing of an artifact frequency component with true respiration component in the TF spectrum. Another common reason for derailed particle filter output was the diminishing RR spectral component for a short while. Both conditions prompted the response of particle filter ultimately to follow some reasonably well-established erroneous path, commonly a low-frequency region characterized by autonomous nervous system (ANS) regulation patterns.

Despite the possibility of occasional derailment of the particle filter, it clearly improves the results compared to picking the maximum component of the spectra, as observed in Table 1. In the tested data, there were only a limited number of cases in which the particle filter declined the performance compared with the extraction of the spectral maximum. We argue that in these cases the other frequency components were distributed so to confuse the performance of particle filter which relies on the evaluation of more than a single frequency component. Results obtained with the theoretical case of being able to always choose the frequency component that is closest to the correct respiration frequency show that there is room for improvement in the selection process, that is, configuration of the particle filter. These results also demonstrate the benefit of all the evaluated synchrosqueezing methods compared with the STFT.

Recall values shown in Table 1 that represent the percentage of the obtained RR output values from the total signal duration are affected by the earlier explained diminished input vectors for particle filtering. Lack of appreciable state vector magnitude resulted in minor periods of missing RR update (empty state vectors) and in rare instances resulting in spreading of the particles until a new frequency component appeared in the vicinity of any particle, increasing the particle weight over the threshold value of 1/*N*. Notably, concentrated modes along the synchrosqueezed TF planes as demonstrated in Figure 2 appeared to preserve mode search better than the smeared presentation of STFT, where the particle filter may have weakened due to lack of distinctively dominant mode regions. VSST and FSST exhibited extraordinary robustness in avoiding recalibration from empty state vectors. However, as can be seen in Table 1, their overall performance was worse than in case of WSST.

## 4. Discussion

FSST and VSST resulted in focused representations of oscillatory modes in TF spectra and particle filtering performed robustly and had high recall values with these methods, that is, their state vectors were rarely empty meaning that at least some of the particles surpassed the 1/*N* weight threshold. All of the proposed TF representation require a priori design variables to provide accurate results from the measurements. It is currently unclear how the variables should be chosen to accommodate the widest range of individuals. Further research, especially in novel methods such as VSST, which benefits from high recall value, may present some improvements. In addition, the fact that WSST has clearly poorer recall value may imply that VSST, in its presented form, is well comparable to WSST in performance. Notably, STFT performed relatively well in our approach, but the results show clear improvement when characteristic features of STFT and inherent assumptions (data stationarity) were discarded by using synchrosqueezing methods. Smeared modes in the STFT spectra assured that there was less deviation in particle filter movement on specific modes, but more ambiguity in establishing the actual RR frequency. Low recall value of STFT with particle filter indicates that there were more transient changes with 5 greatest frequency components in magnitude which particle filter resampling could not follow rapidly enough.

The choice of a priori values for the particle filter, including the properties of the noise for preventing degeneracy, is important for the desired operation of the filter and these values should be chosen carefully. In one hand, the filter may rapidly and blindly follow the assumed dominant mode in the TF representation, and in the other hand only a slow-varying trend of the signal. Accordingly, such values were chosen in the study that were deemed the optimal for each transform and the results are given according to these parameters. One should note that WSST produces wave-like oscillations as output, which are likely to derail particle filter in following the true respiration trend of the TF modes.

One of the major advantages of the synchrosqueezing spectral estimates is the consistency they often provide. We have obtained mean absolute and median error of 2.699 bpm and 1.494 bpm, respectively, with the same dataset by using AR-modeling of low-pass filtered PPG signal and particle filtering [12]. A large number of algorithms with various approaches have been assessed with this dataset in [3,4], where comparison for percent number of results falling at or below the error of 2 breaths (CP_2_) has been given. In particular, synchrosqueezing provided improvement with respect to numerous incorrectly deduced trends extracted by the AR modelling but on the other hand, also shared some of the erroneous trends. This implies that in some cases, modulation properties induced by respiration dynamics are complex and the subtle details of this behavior are not clear. Due to differences in refresh rate of outputs and considering RR reference and PPG signal quality criteria, the results in Table 1 are not directly comparable to [3,4]. However, CP_2_ of the proposed synchrosqueeze-particle filter -cascades were around 58 to 64% and thus perform well among PPG signal algorithms, which were reported to mostly have CP_2_ values at or under 60%, with one distinct method, using a fusion technique, performing at around 71.5% [3]. In contrast to [3,4], we have used whole PPG signal data without removing low-quality waveforms.

Factors affecting the robustness of the proposed methods relate to the extraction of appropriate frequency modes. Small variations caused by measurement artefacts smear the distinction between the frequencies, resulting in ambiguity when determining the correct one. In the absence of a specific modulation component, the proposed spectra may not represent RR as the greatest spectral component during the entire signal. However, particle filtering may be implemented to overcome this difficulty and it is flexible enough to perform on all TF representations. Certain limitations in assessing resolution characteristics, especially as they appear for STFT, may be overcome by synchrosqueezing providing additional benefits for the high-resolution extraction of intrinsic modes, that is, frequency carriers in TF spectra. Therefore, we propose the use of synchrosqueezing methods instead of linear methods, notably STFT or continuous wavelet transform (CWT). In the PPG-derived RR analysis, stationarity is a fundamental assumption that seldom holds in practice. Accordingly, the use of FFT is not a practical approach, especially for longer signal segments when studying respiration dynamics.

The results presented are obtained with data acquired from young subjects at rest and cannot be generalized to, for example, exercise RR estimation. Also, particle filtering includes randomness, which may slightly deviate the results we have presented, although this does not change our conclusions. It is currently unclear how the various parameters should be chosen to accommodate the RR emergence in different PPG measurement environments due to variations in population. Future work should concentrate on finding the compromise values at these low frequency bands of PPG signals. At low-frequency spectra the tracking algorithms, such as particle filter, should be made ‘smart’ in the sense of RR extraction from artefact modes. Combining the improvements of the previously mentioned may further illustrate the potential that this method may have. At current, we have shown that WSST and VSST have both the robustness and the accuracy to be of interested in this field of physiological sensing.

## 5. Conclusions

We have proposed the use of amplitude variability of the PPG signals to estimate RR with the assistance of TF reassignments and a particle filter. In addition to the enhanced signal mode distinction that synchrosqueezing reassignment methods provide compared to STFT, the trend given by these signals appears to provide more consistent results with the reference than the strictly morphologically tracked features of intrinsic mode ridges, the greatest magnitude frequencies, which fluctuate in high time resolution. In the test subjects examined, the reference data was often consistent with highly featured spectrograms provided by synchrosqueezing.

In the future, we are urged to study more accurate RR estimation algorithms for the PPG signal. For instance, the fusion of modulation properties may provide a more dynamic and representative signal than a single modulation source. Further studies of second-order synchrosqueezing, especially when introduced in wavelet methodology, may benefit the development of such modulation extraction protocols. Particle filtering includes several potential properties that may be tuned to fit the algorithm better for the application. Different resampling methods and likelihood functions represent obvious steps for further studies.

Combining the properties of the baseline and rate variability with the amplitude information could provide additional fusion options, which we have disregarded here. We also expect new approaches to PPG instrumentation, for example exploiting variable wavelengths of light, to bring new dimensions for the future algorithms and TF spectra analysis. Also evaluating the proposed method with signals obtained using other sensing methods is an interesting target for future research. While fairly simple algorithms are already able to provide relatively good performance with impedance pneumography signals for most of the time [27], it can be assumed that the proposed method is also able to handle the cases that have otherwise been problematic.

## Figures and Tables

**Table 1:** Mean, mean absolute, median absolute errors, and the root-mean square errors of the studied methods in breaths per minute. CP_2_ represents the percentage of results falling within 2 bpm margin when compared with RR reference. Max.1Spectra represents results if RR was estimated by taking greatest magnitude component of the spectra, Max.5Spectra if out of 5 greatest components, one closest to reference would be chosen. Standard deviations of each value between subjects have been given in parenthesis. The result produced by best performing method in each metrics is shown with green background.

Method: ↓ Error: →	Mean Absolute	Median	Mean	RMS	Recall	CP_2_	Max.1Spectra	Max.5Spectra ^1^
FSST	2.88 (0.85)	1.33 (0.81)	−2.01 (3.35)	4.28 (3.42)	99.8% (0.65%)	60.9% (27.6%)	4.19 (3.02)	1.17 (1.22)
STFT	3.00 (1.27)	1.04 (1.13)	−2.34 (3.41)	4.30 (2.65)	91.3% (16.9%)	59.4% (30.4%)	3.80 (3.21)	3.03 (2.91)
VSST	2.49 (0.97)	1.19 (0.59)	−1.58 (2.31)	3.61 (2.48)	99.9% (0.18%)	64.1% (24.9%)	4.20 (3.01)	1.04 (0.95)
WSST	2.33 (0.89)	1.15 (0.46)	−0.76 (2.55)	3.68 (1.09)	97.1% (6.9%)	64.3% (25.6%)	3.62 (2.22)	1.54 (1.12)

^1^ Hypothetical scenario.
